# The impact of national vaccination policy changes on influenza incidence in the Netherlands

**DOI:** 10.1111/irv.12366

**Published:** 2016-02-02

**Authors:** Scott A. McDonald, Liselotte van Asten, Wim van der Hoek, Gé A. Donker, Jacco Wallinga

**Affiliations:** ^1^ Centre for Infectious Disease ControlNational Institute for Public Health and the EnvironmentBilthovenThe Netherlands; ^2^ NIVEL Primary Care DatabaseSentinel PracticesUtrechtThe Netherlands

**Keywords:** Influenza, influenza‐like illness, statistical model, the Netherlands, vaccination policy

## Abstract

**Background:**

We assessed the impact of two major modifications of the Dutch National Influenza Prevention Programme – the introduction in 1997 of free‐of‐charge vaccination to persons aged ≥65 years and to high‐risk groups (previously only advised, and not free of charge), and the lowering of the eligible age to 60 years in 2008 – on the estimated incidence of influenza infection leading to influenza‐like illness (ILI).

**Methods:**

Additive negative‐binomial segmented regression models were fitted to ILI data from GP sentinel surveillance in two‐eight‐season intervals (1993/4 to 2000/1, 2004/5 to 2011/12, comparing pre‐ and post‐policy‐change periods within each interval), with laboratory virological reporting of samples positive for influenza or other ILI‐causing pathogens as covariates.

**Results:**

For the 2008 policy change, there was a significant step decrease in influenza contribution considering all ages (=−111 per 100 positives; 95% CI: −162, −65·0), <60 years and 60–64 years age groups (*B *=* *−92·1 per 100; 95% CI: −134, −55·5; *B *=* *−5·2; 95% CI: −10·3, −1·2, respectively). There was no evidence for a decrease associated with the 1997 policy change targeting the ≥65 years age group.

**Conclusions:**

In the Netherlands, a 56% reduction in influenza contribution was associated with the 2008 policy targeting 60–64 year‐olds, but there was no effect of the earlier policy targeting ≥65‐year‐olds, for whom vaccination coverage was already rising before the policy change.

## Introduction

Two major modifications to influenza vaccination policy in the Netherlands have occurred within the past two decades. With the introduction in 1997 of the Dutch National Influenza Prevention Programme, a nation‐wide primary care prevention programme, influenza vaccination is offered free of charge to a number of medical high‐risk groups and persons aged 65 and older irrespective of a medical indication,^1^, ^2^ thus providing free vaccination to persons ≥65 years starting with the 1997/98 influenza season. Before 1997, influenza vaccination was only advised for these groups and was not free of charge. Patients are invited by their general practitioner (GP) for vaccination in October or November, before the start of the influenza season. This population‐based promotion campaign supported GP practices through fees‐for‐service and the provision of software to assist in patient selection and invitation. In 2008, the minimum age for which vaccination was offered free of charge to non‐medical risk groups was lowered to 60 years^3^ (thus making free vaccination available to persons ≥60 years from the 2008/09 influenza season onwards). Given the major investment and the large proportion of the population involved, it is of interest to estimate the impact of both prevention initiatives on influenza incidence subsequent to their introduction.

One approach for addressing this objective is to apply analysis methods to the time series of ILI consultations from sentinel GPs to directly assess the impact of policy change(s) on ILI rates. This has the advantage of simplicity and allowing comparison between countries with similar ILI sentinel surveillance systems.^4^ If, instead, the goal is to measure the association between vaccination policy and *influenza* incidence, we must implement a method to attribute ILI cases to the set of possible underlying causes. Such methods include regression modelling approaches in which the circulation of influenza and other viruses potentially responsible for ILI is taken into account,^5^, ^6^ and methods that restrict the periods of ILI analysis to influenza‐active weeks (the ‘rate‐difference’ method).^7^, ^8^ The latter method, although shown to be effective for addressing certain questions, implicitly assumes that all ILI cases during influenza‐active weeks (usually incorporating the peak of an ILI case curve) are attributable to influenza. Complicated cases of respiratory disease developing in patients with ILI symptoms may not necessarily be caused by influenza even in times of peak ILI.^9^ It is therefore of value to estimate influenza‐attributable ILI taking into consideration the co‐circulation of other ILI‐causing pathogens.

The goal of the current research is to assess the impact on influenza‐attributable ILI consultations associated with the two above‐mentioned national vaccination policy changes in the Netherlands using an interrupted time‐series design^10^, ^11^ to compare pre‐ and post‐intervention periods. To take into account the fact that only a proportion of ILI patients have true influenza infection, we integrate segmented regression with a previously developed linear regression method for estimating the contribution of influenza to ILI, which exploits the relationship between seasonal variability in ILI and temporal variation in the circulation of influenza and other ILI‐causing pathogens.^5^, ^12^ Specifically, we wished to establish whether the rate of GP consultations with influenza‐attributable ILI has decreased subsequent to changes in national vaccination policy, and if so, whether the age groups targeted by the policy were affected more than other age groups.

## Methods

### Data sources

#### GP consultation rates for ILI from sentinel surveillance

The sentinel surveillance system of NIVEL Primary Care Database of the Netherlands was established in 1970; coverage is national and is representative by age, sex and population density. Approximately 40 sentinel GP practices representing about 0·8% of the Dutch population^13^ contribute data on consultations for ILI. An ILI case is defined as acute onset of disease, accompanied by a raised rectal temperature of >38°C, and at least one of the following symptoms: cough, coryza, sore throat, frontal headache, retrosternal pain or myalgia. We extracted the weekly numbers of ILI consultations and sentinel GP catchment population sizes for the seasons 1993/94 through 2011/12, where a season is defined as the period from week 40 of a given year through the end of week 39 of the following year. Data were stratified by age group (<60, 60–64 and 65+ years) to match the age groups targeted by the two vaccination policy changes. Over these 19 influenza seasons (1993/94 through 2011/12), a total of 45 175 ILI consultations were registered (mean of 2378 consultations per season). A total of 37 328, 2147 and 5700 consultations were reported for the <60, 60–64 and 65+ years age groups, respectively.

**Figure 1:**
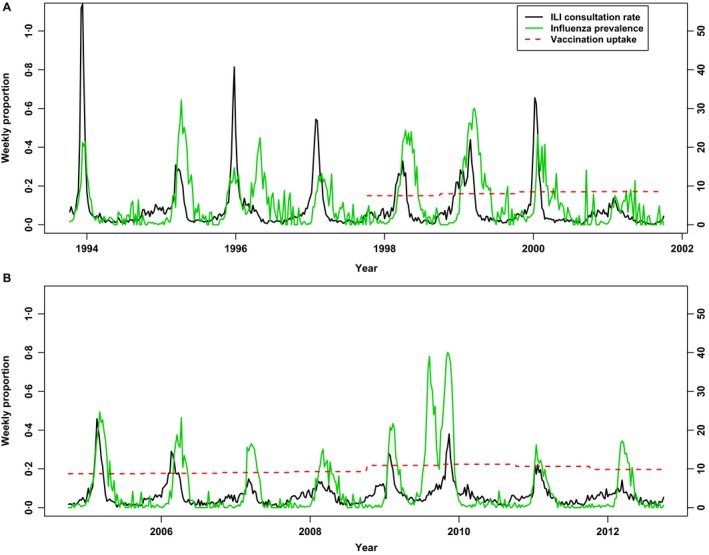
Weekly ILI consultation rates (from sentinel GP practices), influenza prevalence (estimated from weekly laboratory virological reporting of positive test results of influenza and the other five ILI‐causing viruses considered) and annual seasonal influenza vaccination uptake (all ages), for the eight‐season period 1993/94 to 2000/2001 (A) and for the eight‐season period 2004/05 to 2011/2012 (B). ILI consultation rate (per 10 000) is shown on the right axis of each plot.

Annual estimated influenza vaccination coverage is routinely recorded by NIVEL Primary Care Database, a nationally representative network of GPs^14^; Figure 1 plots coverage by year. Vaccination coverage was unavailable from this source for the years before 1996.

#### Laboratory virus surveillance weekly reporting

Routine weekly data on positive laboratory results for a range of pathogens, including pathogens that can cause ILI symptoms, have been reported since 1989. Between 17 and 21 laboratories in the Netherlands submit weekly reports to a centralised database (the Weekly Surveillance System of the Dutch Working Group on Clinical Virology). For the current study, we considered PCR‐confirmed positive samples for the following viral and bacterial agents: influenza A/B, respiratory syncytial virus (RSV), rhinovirus, parainfluenza and *Mycoplasma pneumoniae*. We extracted data from 1993/94 through 2011/12, defining seasons according to the same definition as for the sentinel practices ILI data.

Given the relatively small variation in the level of vaccination coverage over the analysis time intervals (Figure 1; see below), the effects of the policy changes on susceptibility to infection – and thus on incidence – are also anticipated to be small. We can estimate the expected drop in influenza incidence associated with the later (2008) policy change based on published figures on vaccination uptake and effectiveness. Vaccination uptake (all ages) was approximately 18·0% over the four‐season period preceding the change, increasing to approximately 21·3% following the change (Figure 1B). On the basis of 2010/11 data from seven European sentinel networks, an effectiveness of 42·3% in vaccination target groups was recently estimated, for all influenza subtypes combined.^15^ Assuming this value of 42·3% for vaccine effectiveness (and assuming no impact on transmission) and a rise in vaccination uptake from 18·0 to 21·3%, a decrease in incidence of [(0·213–0·180) × 0·423] = 1·40% following the policy change would be anticipated. To detect such a small expected incidence decrease, we adopted statistical modelling methods.

### Data analysis

Our analysis goal was to estimate the contribution of influenza to weekly ILI consultations, adjusting for the activity of a defined set of candidate pathogens other than influenza that could also give rise to ILI symptoms (namely RSV, rhinovirus, para‐influenza and *Mycoplasma pneumoniae*). For simplicity, we assumed additivity: only a single pathogen could be responsible for ILI in a given patient in a given week. Our approach involved specifying a statistical model in which the seasonal variability in ILI consultations is explained by temporal variation in the incidence of various ILI‐causing pathogens. By fitting a linear regression model to a relevant outcome such as syndromic surveillance, hospitalisation or mortality data, the proportion of the outcome attributable to influenza can be determined after adjusting for the co‐circulation of other pathogens.^5^, ^12^, ^16^, ^17^, ^18^

We specified *a priori* two‐eight‐season analysis intervals (1993/4 to 2000/1 and 2004/5 to 2011/12) for evaluation of the impact of the two policy changes; this allowed comparison of the four seasons before and after each policy change. The week level of granularity was used for all data sources and regression analyses. Separate negative‐binomial regression models were fitted to ILI data for all ages aggregated together, for persons under 60 years, persons aged 60–64 years and the 65+ years age group. As the weekly laboratory surveillance data were not available stratified by age, the same reported (total) numbers of positive samples were used as covariates in each age group‐specific regression model.

The basic model is:

(1) $$E\left(Y_{i}\right)=B_{0}+\sum_{j=1}^{n}B_{j}X_{ij},$$

where *i* indexes week number and where *j* indexes the causal pathogen, *Y*_i_ is the observed number of ILI consultations in week *i*,* B*_0_ is a constant (or intercept) term, and *X*_*i,*1_…_5_ are the weekly laboratory reported numbers for RSV, rhinovirus, para‐influenza, *Mycoplasma pneumoniae* and influenza.

We considered the impact of possible lag time between the weekly laboratory surveillance data with respect to the weekly ILI data, as laboratory data surveillance probably reflect more testing of hospitalised than GP patients, and used model‐fitting methods to establish whether the pathogen counts *X*_1_…_5_ should be entered into the regression model as lagged variables or not.^18^ Plots of the time series of the weekly numbers of influenza positive samples from laboratory surveillance overlaid with the weekly numbers of influenza positive samples from virological testing routinely performed on sampled ILI patients from the sentinel practices indicated a delay of the seasonal peaks (Figure S2). We successively compared models incorporating 0‐, 1‐, 2‐ and 3‐week lags between the sentinel practices and laboratory data, selecting the lag time that represented the largest improvement in model fit over a 0‐week lag according to the Akaike information criterion (AIC). An AIC difference >2 was deemed an improved fit. Equation (2) provides the general form of an additive regression model including *n* pathogens as covariates:

(2) $$E\left(Y_{i}\right)=B_{0}+\sum_{j=1}^{n}B_{j}X_{\left(i-\text{lag}\right)j}$$

### Segmented regression analysis

To evaluate the impact of change in vaccination policy, we combined the above additive model with segmented regression analysis.^11^ The interrupted time‐series design is an effective and robust method for evaluating any change in an epidemiological outcome measure that is associated with an intervention, because both the pre‐intervention level and any secular trend in the outcome can be taken into account.^10^ For the analysis of the impact on estimated influenza incidence associated with the 1997 and 2008 policy changes, we compared the four‐season periods before and after the intervention time point, which was assumed to occur between week 39 and week 40 (typically the beginning of October; this change point is consistent with the invitation of patients by their GP to receive the vaccine in October and November, and the definition of the start of the ILI season for data analysis). The regression model above (Eq. (2)) was therefore replaced with Eq. (3) below, and was fitted to the weekly data on GP consultations for ILI:

(3) $$E\left(Y_{i}\right)=B_{0}+\sum_{j=1}^{5}B_{j}X_{\left(i-\text{lag}\right)j}+B_{6}\left({\text{Time}}_{i}\cdot X_{\left(i-\text{lag}\right)5}\right)+B_{7}\left({\text{Period}}_{i}\cdot X_{\left(i-\text{lag}\right)5}\right)+B_{8}\left({\text{TimePost}}_{i}\cdot X_{\left(i-\text{lag}\right)5}\right).$$

In this segmented regression model, *Y*_i_ and *X*_*i,*1_…_5_ represent the same weekly data as described for Eq. (1). As before, *i* indexes week number and *j* indexes the causal pathogen, with *j *=* *5 for influenza. Time encodes the ordinal week number counting from the start of the eight‐season analysis interval; TimePost encodes the number of weeks following the change point; and *Period* is a binary variable with ‘0’ and ‘1’ indicating pre‐ and post‐policy change periods, respectively, within the eight‐season interval analysed. The following interaction terms were also included: the *B*_6_ term encodes the secular trend in the influenza coefficient (*B*_5_); *B*_7_ encodes a level change in the influenza coefficient (*B*_5_) in the post‐ compared with pre‐policy change periods; and *B*_8_ encodes any change in trend in the influenza coefficient (*B*_5_) in the post‐ compared with the pre‐change period. The outcome of interest – a step change in the proportion of influenza‐attributable ILI cases between periods – is indicated by a significant *B*_7_ coefficient. All regression models were fit using R statistical computing software.^19^ Standardised residual plots were examined, and no patterns in error variance were apparent. The autocorrelation function of the residuals was also examined and Durbin–Watson test conducted; there was positive autocorrelation at lags 1 and 2 apparent. No adjustment was made, as inclusion of autocorrelation terms may compete for variance associated with the co‐circulating pathogens,^20^ but standard error estimates may be biased downwards.

## Results

The ILI consultation rate time series from the sentinel GP practices, together with the prevalence of influenza from weekly laboratory surveillance (in relation to the total reported positive samples for influenza and the other co‐circulating pathogens considered), are shown for the two analysis intervals separately (Figure 1). Results of the segmented negative‐binomial regression analysis assessing the impact of the vaccination policy change in 1997 are provided in Table 1. For convenience, regression coefficients that are presented are multiplied by 100 and thus indicate, for example, ILI cases per 100 positive influenza laboratory reports. Lags of two and one weeks for the laboratory surveillance data were determined to provide the best fit for the earlier and later analysis intervals, respectively. The estimated contribution to ILI from influenza virus infection, adjusting for the circulation of RSV, rhinovirus, para‐influenza and *Mycoplasma pneumoniae,* was then compared across pre‐ and post‐policy change periods via tests of interaction terms (Table 1).

**Table 1:** Segmented regression modelling results, in which separate additive negative‐binomial models were fitted to ILI consultations for each eight‐season interval and age category

Covariate	All ages		<60 years		60–64 years		65+ years	
	*B* a	95% CI	*B* a	95% CI	*B* a	95% CI	*B* a	95% CI
Interval 1993/04 to 2000/01								
Constant^b^	**716**	**455, 1006**	**551**	**329, 799**	**69·5**	**38·0, 105**	**77·3**	**29·8, 130**
Influenza activity	**440**	**343, 555**	**379**	**295, 478**	**25·5**	**17·6, 34·4**	**42·6**	**31·5, 56·4**
Initial period trend for influenza^c^	−**1·1**	−**1·9,** −**0·33**	−**1·0**	−**1·7,** −**0·33**	−**0·08**	−**0·15,** −**0·01**	−0·05	−0·16, 0·05
Level change for influenza^c^	22·5	−86·0, 128	14·4	−76·8, 102	5·5	−4·1, 15·0	1·5	−14·8, 17·6
Latter period change in trend for influenza	0·83	−0·19, 1·9	0·83	−0·03, 1·7	0·03	−0·06, 0·12	−0·01	−0·15, 0·14
Interval 2004/05 to 2011/12								
Constant^b^	**513**	**304, 732**	**392**	**226, 567**	**40·0**	**8·4, 74·2**	**98·9**	**33·5, 170**
Influenza activity	**197**	**158, 242**	**161**	**129, 198**	**7·7**	**4·9, 11·3**	**31·8**	**23·4, 42·3**
Initial period trend for influenza^c^	−0·21	−0·57, 0·16	−0·15	−0·45, 0·14	−0·006	−0·04, 0·03	−**0·09**	−**0·17,** −**0·01**
Level change for influenza^c^	−**111**	−**162,** −**65·0**	−**92·1**	−**134,** −**55·5**	−**5·2**	−**10·3,** −**1·2**	−8·7	−20·0, 0·48
Latter period change in trend for influenza	0·08	−0·30, 0·46	0·04	−0·27, 0·35	−0·0006	−0.03, 0·03	0·08	−0.001, 0·16

CI, confidence interval.

a Regression coefficients are multiplied by 100 and thus reflect ILI cases per 100. Coefficients are adjusted for activity of the other four pathogens in the model (RSV, rhinovirus, parainfluenza, *Mycoplasma pneumoniae*).

b The constant, or intercept term, accounts for ILI cases not attributed to any of the pathogens considered.

c Fitted *time* × *influenza* and *period* × *influenza* interaction terms capture changes in the contribution of influenza activity as a linear function of time and as a binary function of period (before versus after the effective date of vaccination policy change), respectively. Boldface indicates conventional significance: *P *<* *0·05.

**Figure 2:**
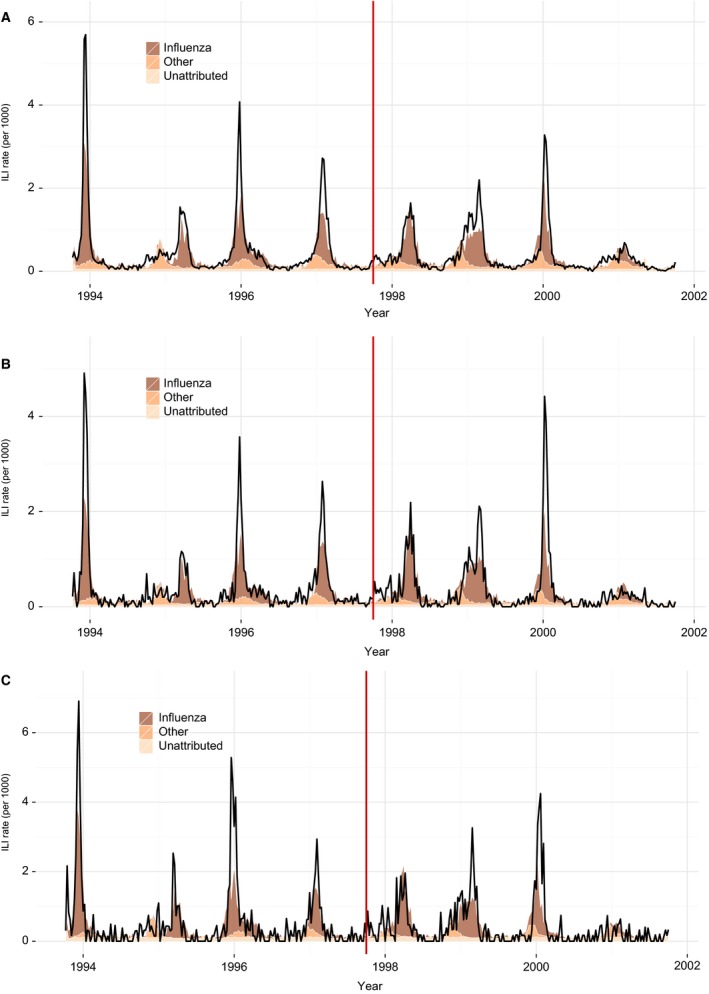
ILI consultation rates and fitted additive contributions from influenza and the other four circulating pathogens, for the eight‐season period 1993/94 to 2000/01. Fits to ILI consultation rates are displayed separately for all ages aggregated together (panel A) and for persons 65+ years only (panel B), and for persons 60–64 years only (C).

Weekly ILI consultation rates and fitted values over a period of eight seasons are shown for all age groups aggregated together, for persons aged 60–64 years only and for persons aged 65+ (Figure 2). Considering all age groups, after adjustment for the other four co‐circulating pathogens and for a small decreasing secular trend in influenza contribution (Table 1), there was no evidence for a significant decrease in the contribution of influenza to ILI consultations in the post‐policy change period (*B *=* *22·5 per 100*; P *=* *0·69). There was also no evidence for any change in influenza contribution when restricting analysis to the <60, 60–64 or ≥65 years age groups (*P*‐values of 0·77, 0·23 and 0·86, respectively).

**Figure 3:**
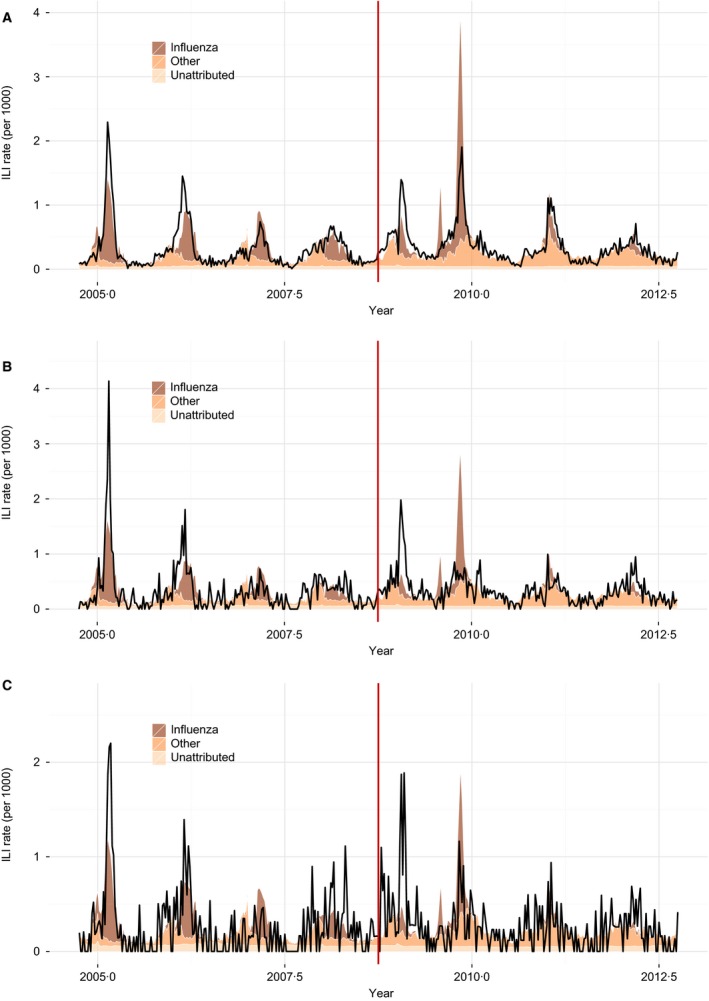
ILI consultation rates and fitted additive contributions from influenza and the other four circulating pathogens, for the eight‐season period 2004/05 to 2011/12. Fits to ILI rates are displayed separately for all ages aggregated together (panel A), for persons 65+ years only (B) and for persons 60–64 years only (C).

The same approach was applied to evaluate the impact of the 2008 policy change in which the eligible age for free of charge vaccination was lowered to 60 years. Weekly ILI rates and fitted values over an interval of eight seasons are shown for all age groups aggregated together, for the age group 60–64 years only and for persons aged 65+ years (Figure 3). Considering all ages, after adjustment for the other four circulating pathogens and a small decreasing secular trend in influenza contribution, there was a significant step decrease in influenza contribution post‐policy change (*B *=* *−111 per 100; 95% CI: −162, −65·0). This 56% reduction in influenza contribution translates to a predicted average annual number of 951 ILI consultations attributable to influenza infection (50·9% of the total average annual ILI consultations) in the pre‐policy change period, and an average annual number of 860 influenza‐attributable ILI cases (40·5% of the total average annual ILI consultations) in the post‐change period. Results differed by age group. A significant step decrease in influenza contribution post‐policy change was observed for the <60 years age group (*B *=* *−92·1; 95% CI: −134, −55·5) and the 60–64 years age group (*B *=* *−5·2; 95% CI: −10·3, −1·2), but not for the 65+ years age group (*P *=* *0·095).

## Discussion

Using a statistical modelling approach, we found a step decrease in the adjusted contribution of influenza to the ILI consultation rate associated with the second policy change investigated (in 2008) only, both when aggregating all ages together and when restricting to the target age group of persons aged 60–64 years. In terms of influenza‐attributable ILI rates, there was no evidence that the 65+ age group benefitted from changes in vaccination policy, in either interval investigated. However, any impact on ILI in the earlier interval may not have been detectable because vaccination coverage in this age group was already high (76% in 1996/97, increasing to 79% in 1997/98 according to data from the NIVEL Primary Care Database). Data from an annual national survey on a random sample of the population corroborate the gradually increasing vaccination uptake in this age group (11% in 1996/97; 13% in 1997/98) (Statistics Netherlands^21^), and other survey‐based data indicated a rise in vaccination uptake among high‐risk groups between 1991 and 1996.^22^ Although data are limited regarding practice prior to the introduction of the Dutch National Influenza Prevention Programme, guidelines have been in existence since 1993 and earlier; GPs were not specifically advised to offer vaccination to persons aged 65 years and over who did not also have a medical indication.^23^ However, the guidelines advocated applying criteria broadly in the elderly; thus, these patients could be invited for vaccination at the discretion of their GP.^24^ Nonetheless, it is clear that evaluation of the impact of vaccination programmes on the incidence of influenza cannot be conducted based on vaccination coverage alone, or on assessment of their implementation in GP practices.^25^

In the regression analyses restricted to persons aged <60 years (Table 1), a step decrease in influenza contribution to ILI was observed relating to the 2008 vaccination policy change only. This may have been due to a reduction in transmission from the older, vaccinated age groups, or to possible improved vaccination effectiveness among members of high‐risk medical groups in the under 60‐year‐olds. We do not have the required data to confirm or reject either possibility.

The pH1N1 influenza epidemic is considered to have begun in week 41 (October) of 2009 in the Netherlands, lasting for 10 weeks.^26^ During this period, there was a clear increase in the weekly number of influenza positive samples reported by the laboratory surveillance system (Figure 1B), which may reflect both increased influenza activity and enhanced surveillance, which was implemented in late April 2009^27^ and continued through late May 2010. Fitted model coefficients for influenza and the influenza × period interaction term may have been unduly influenced by this peak. However, our segmented regression analysis findings for the 2008 policy change did not appear to be affected by the presence of the pandemic year; the significant step decrease in influenza contribution in the post‐policy change period remained when fitting a regression model to the same interval, but with the pandemic season 2009/10 excluded (Figure S1).

The power to detect across‐period differences in influenza‐attributable ILI associated with the two changes in policy depends on both vaccination uptake and vaccine efficacy. If seasonal vaccination was completely ineffective, then no difference in the influenza‐attributable proportion between periods would be expected. If we consider the 2008 policy change only, our model inferred a decrease from 951 (pre‐policy change) to 860 (post‐change) annual average influenza‐attributable ILI consultations in the sentinel practices; this corresponds to a decrease in influenza incidence of 10·4%. Although this model‐predicted decrease is higher than our calculation of the expected drop in incidence based on vaccination uptake and effectiveness (i.e. 1·4%; see Methods, above), it is roughly comparable because the effect of increased vaccination coverage on transmission (and thus on influenza incidence) is nonlinear, and depends on the age groups vaccinated and their contact patterns.

There are several limitations with the current statistical modelling approach. First, by fitting a single, time‐invariant regression coefficient for each circulating pathogen, we make the assumption that the proportion of all ILI consultations that is attributable to each pathogen is constant across the study period. Hence, influenza contribution would be nonzero during weeks with nonzero ILI that may in reality be completely caused by another pathogen(s). We minimised this effect by taking short 8‐year intervals rather than analysing the entire long time series. Second, we assume that the entirety of the seasonal variation in ILI consultations is explainable by temporal variability of five pathogens, with a constant term representing ILI not attributable to any of the set of five. Although the omission of other factors (e.g. the circulation of other, non‐monitored pathogens, climatic factors such as temperature, etc.) means that a suboptimal fit is achieved, the model is arguably adequate for answering the current research question. Incorporation of seasonally varying climatic variables into the current model has the disadvantage of adding complexity to the interpretation of the findings, because seasonal variance inherent in such variables can compete with temporal variation in the activity of the other ILI‐causing pathogens in the regression model.^28^

Third, all pathogens’ contributions to ILI were treated as invariant by age group – although age dependence is plausible – because age‐specific laboratory surveillance data were unavailable. Finally, the time invariance in laboratory surveillance for influenza and the other four co‐circulating pathogens was assumed; that is, there were no changes in testing or reporting protocols, or improvements in laboratory testing methodology, that could affect the probability of reporting.

Future extensions to the current work could take into consideration the actual vaccination coverage per age group and season, to assess the impact of vaccination – as opposed to changes in policy – on estimated influenza incidence. Note that the current work does not investigate the impact of vaccination policy changes on the risk of developing severe complications,^29^ to which hospitalisation (e.g. admission frequency, length of stay)^30^ or mortality data^8^, ^18^, ^30^ may be more sensitive. We also do not address the impact of vaccination policy change on cases with less severe symptoms (and therefore do not consult their GP).

In summary, the combination of surveillance data sources – ILI data from GP sentinel surveillance and routine weekly laboratory virological reporting of ILI‐causing pathogens – revealed evidence for a decrease in influenza‐attributable ILI associated with the 2008 policy change in which the eligible age for free of charge vaccination was lowered to 60 years, suggesting that this policy change was successful in reducing the incidence of influenza in the targeted age group. Our method did not detect any association between the introduction in 1997 of free‐of‐charge vaccination for ≥65 years and a change in the estimated incidence of influenza, but vaccination uptake was already rising preceding the 1997 policy change. The current methodology may prove useful to other countries interested in addressing similar questions regarding assessing the effectiveness of national prevention measures, for the situations where only syndromic surveillance outcome data are available.

## Supporting information


**Figure S1.** ILI consultation rates and fitted additive contributions from influenza and the other four circulating pathogens, for the eight‐season period 2004/05 to 2011/12, for all ages aggregated together (with the 2009 season excluded in analyses).** Figure S2.** Comparison of the time series of the weekly number of influenza positive samples from laboratory surveillance with the weekly number of confirmed influenza positives from virological testing of a sample of ILI patients from sentinel GP practices, for the 19 seasons 1992/3 to 2011/12. The right axis indicates the weekly number of influenza positives from laboratory surveillance. **Table S1.** Segmented regression modelling results, in which separate additive negative‐binomial models were fitted to ILI patients aged 65+ years, divided into narrower age categories.
