# Supplementary material for: The impact of national vaccination policy changes on influenza incidence in the Netherlands
Source: Influenza Other Respir Viruses. 2016 Feb 2;10(2):76–85. doi: 10.1111/irv.12366 (PMC4746562; doi:10.1111/irv.12366)
Supplement: Supplementary file 1 — Figure S1. ILI consultation rates and fitted additive contributions from influenza and the other four circulating pathogens, for the eight‐season period 2004/05 to 2011/12, for all ages aggregated together (with the 2009 season excluded in analyses). Figure S2. Comparison of the time series of the weekly number of influenza positive samples from laboratory surveillance with the weekly number of confirmed influenza positives from virological testing of a sample of ILI patients from sentinel GP practices, for the 19 seasons 1992/3 to 2011/12. The right axis indicates the weekly number of influenza positives from laboratory surveillance. Table S1. Segmented regression modelling results, in which separate additive negative‐binomial models were fitted to ILI patients aged 65+ years, divided into narrower age categories. [file IRV-10-076-s001.docx]

**SUPPORTING INFORMATION**

**Estimating the impact of national vaccination policy changes on influenza incidence in the Netherlands**

Scott A. McDonald^1^, Liselotte van Asten^1^, Wim van der Hoek^1^, Gé A. Donker^2^, Jacco Wallinga^1^

^1^Centre for Infectious Disease Control, National Institute for Public Health and the Environment, Bilthoven, The Netherlands

^2^NIVEL Primary Care Database, Sentinel Practices, Utrecht, The Netherlands.

As submitted to *Influenza and Other Respiratory Infections*

In a first supplementary analysis, our aim was to establish if there was any influence of the vastly increased number of positive influenza samples reported in laboratory surveillance in 2009 (when laboratory testing was intensified due to the H1N1 pandemic) relative to other years (see Fig. 1B). In the course of fitting this peak (occurring in the latter period, after the change point), the model may have overestimated the contribution of influenza in the earlier period. To evaluate the impact of this occurrence, we refitted the negative binomial regression model to the later eight-season interval (2004/05 to 2011/12), but excluding the pandemic H1N1 season 2009/10 so that there could be no possible influence from the huge peak in positive influenza samples from laboratory surveillance (Fig. S1). The significant step increase in the contribution of influenza post-policy change remained: -95.9 per 100 influenza positive samples (95% CI: -148 to -49.1), which is slightly smaller than observed when fitting the regression model to the entire interval including the pH1N1 season: -111 per 100 (95% CI: -162 to -65.0).

We conducted further supplementary analyses by dividing the 65+ years age-group into narrower groups: 65-69, 70-79, and 80+ years. Results of these additional age-specific negative-binomial regression analyses indicated that there was no evidence for a change in the contribution of influenza to ILI between early and later periods, in both intervals investigated (Table S1). These findings are in agreement with the results treating the 65+ years age-group as a homogenous category.

**Figure S1.** ILI consultation rates and fitted additive contributions from influenza and the other four circulating pathogens, for the eight-season period 2004/05 to 2011/12, for all ages aggregated together (with the 2009 season excluded in analyses).

**
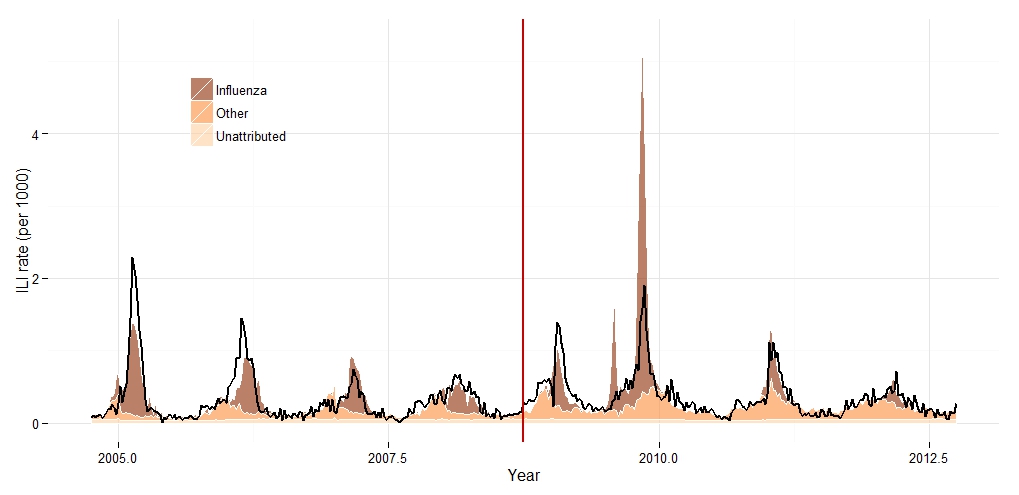
**


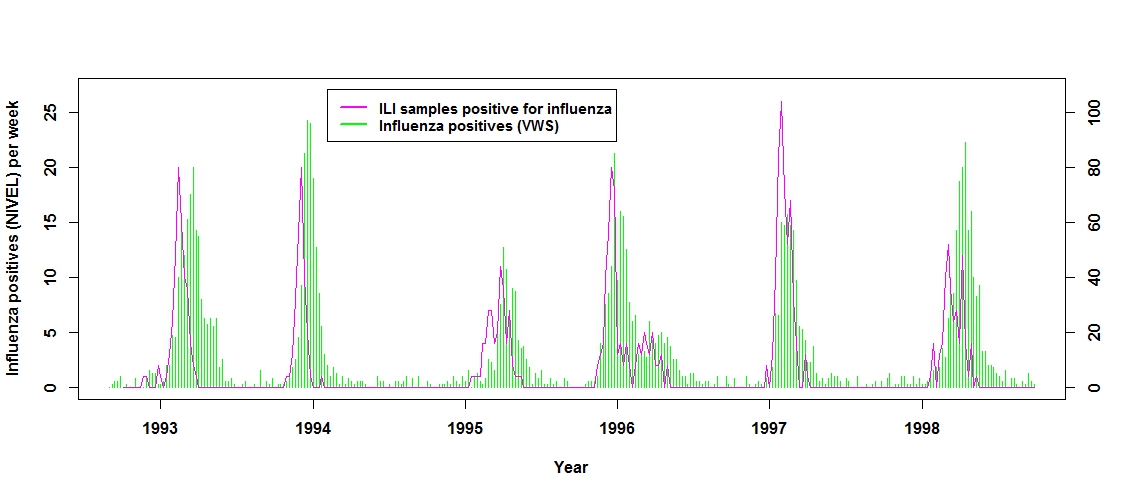
**Figure S2.** Comparison of the time series of the weekly number of influenza positive samples from laboratory surveillance with the weekly number of confirmed influenza positives from virological testing of a sample of ILI patients from sentinel GP practices, for the 19 seasons 1992/3 to 2011/12. The right axis indicates the weekly number of influenza positives from laboratory surveillance.

**
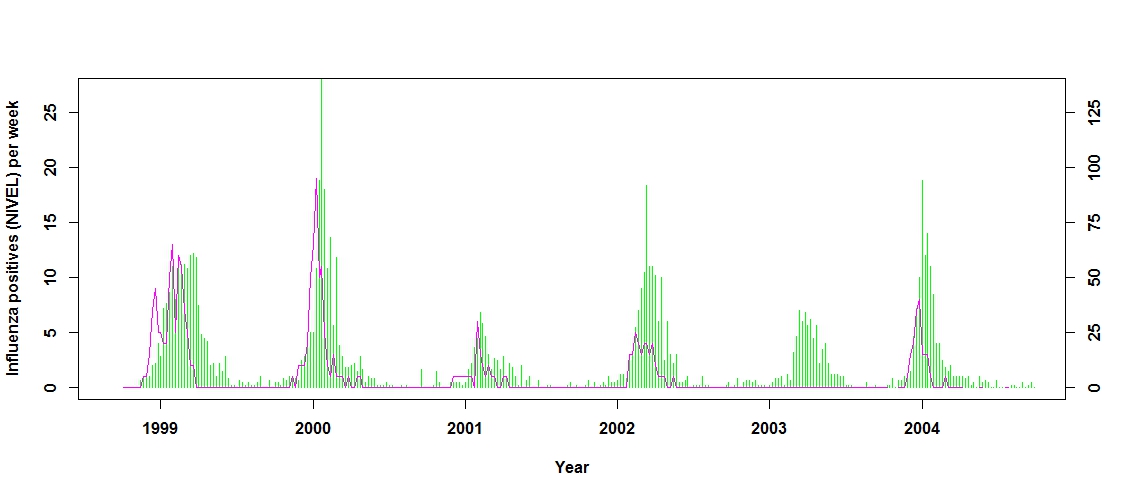

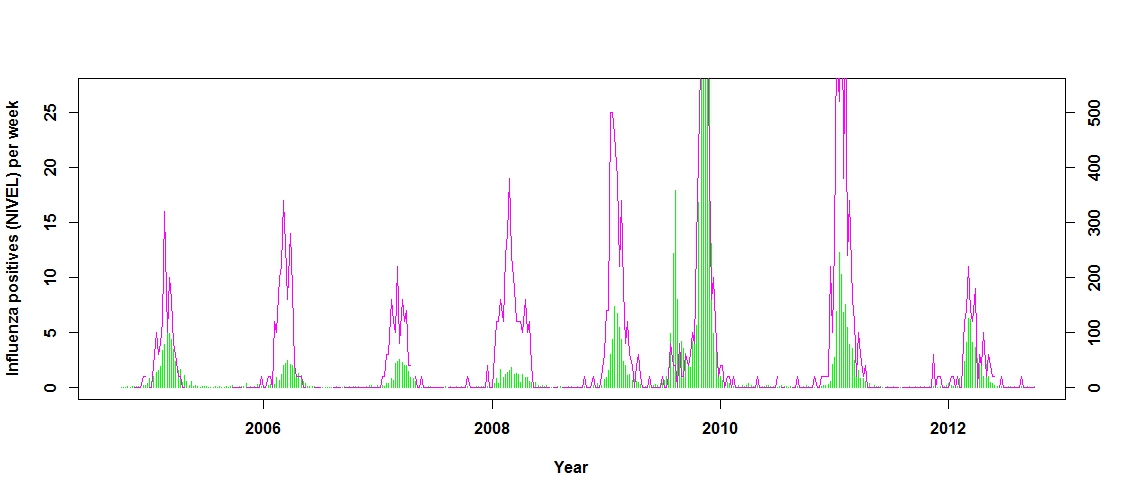
**

**Table S1.** Segmented regression modelling results, in which separate additive negative-binomial models were fitted to ILI patients aged 65+ years, divided into narrower age categories.

65-69 years 70-79 years 80+ years

Covariate *B*† (95% CI) *B*† (95% CI) *B*† (95% CI)

*Interval 1993/04 to 2000/01*

Constant 24.8 (-1.3, 50.9) **42.2 (10.7, 73.8) 13.2 (-5.2, 31.7)**

Influenza activity **15.2 (9.7, 20.6) 24.8 (17.1, 32.4) 7.4 (3.5, 11.3)**

Initial period trend
 for influenza -0.02 (-0.06, 0.02) -0.04 (-0.10, 0.02) -0.009 (-0.04, 0.02)

Level change
 for influenza -0.8 (-7.2, 5.6) 0.8 (-7.8, 9.5) 2.8 (-2.5, 7.9)

Latter period change
in trend for influenza 0.007 (-0.05, 0.07) 0.01 (-0.07, 0.09) -0.01 (-0.06, 0.04)

*Interval 2004/05 to 2011/12*

Constant* 25.2 (-1.9, 55.9) 34.9 (-2.35, 77.1) **49.1 (17.3, 82.7)**

Influenza activity **8.3 (5.7, 11.6)** **15.3 (10.8, 21.2) 9.8 (6.2, 14.5)**

Initial period trend
 for influenza -0.03 (-0.05, -0.002) **-0.06 (-0.10, -0.01)** -0.02 (-0.06, 0.01)

Level change
 for influenza -1.7 (-5.5, 1.3) -2.6 (-8.5, 2.0) -3.1 (-8.7, 1.2)

Latter period change
in trend for influenza 0.05 (-.01, 0.10) **0.05 (.008, 0.09)** 0.02 (-0.02, 0.06)

*Note. CI*=confidence interval. † Regression coefficients are multiplied by 100, and thus reflect ILI cases per 100. Coefficients are adjusted for activity of the other four pathogens in the model (RSV, rhinovirus, para-influenza, *Mycoplasma pneumoniae*). Boldface indicates conventional statistical significance: *P* < 0.05.
